# WNT4 promotes the symmetric fission of crypt in radiation-induced intestinal epithelial regeneration

**DOI:** 10.1186/s11658-024-00677-4

**Published:** 2024-12-26

**Authors:** Jingyang Cheng, Haiyong Wu, Yanmei Cui

**Affiliations:** 1 Guangdong Provincial Key Laboratory of Colorectal and Pelvic Floor Diseases, Guangdong Institute of Gastroenterology, The Sixth Affiliated Hospital, Sun Yat-Sen University, Guangzhou, People’s Republic of China; 2 Biomedical Innovation Center, The Sixth Affiliated Hospital, Sun Yat-Sen University, Guangzhou, People’s Republic of China

**Keywords:** WNT4, Crypt fission, Paneth cell, Epithelial regeneration

## Abstract

**Background:**

Radiotherapy for pelvic malignant tumors inevitably causes intestinal tissue damage. The regeneration of intestinal epithelium after radiation injury relies mainly on crypt fission. However, little is known about the regulatory mechanisms of crypt fission events.

**Methods:**

The effects of WNT4 on crypt regeneration and the symmetry of crypt fission were examined using a mouse small intestinal organoid culture model. Three-dimensional (3D) reconstructed images of organoids were applied to assess the symmetry of crypt fission and Paneth cell localization upon manipulation of WNT4 expression. The effect of WNT4 on the expression of β-catenin target genes was analyzed by real-time quantitative polymerase chain reaction (RT-qPCR). The *in vivo* effect of WNT4 overexpression mediated by adeno-associated virus (AAV) on symmetric fission of crypt was investigated using a radiation-injured mouse model.

**Results:**

WNT4 has a special function of promoting symmetric fission of small intestinal crypts, although it inhibits budding, stemness, and cell proliferation on organoids. WNT4 promotes the correct localization of Paneth cells in the crypt base by regulating the expression of EphB3, thereby promoting the symmetric fission of small intestinal crypts. WNT4 negatively regulates the canonical WNT/β-catenin signaling pathway, and it promotes symmetric crypt fission in a ROR2 receptor-dependent manner. Moreover, in patients and animal models of radiation-induced intestinal injury, we found that the regenerated crypts are irregular in size and shape, Paneth cells are mislocalized, and the expression of WNT4 is decreased while EphB3 is increased. Importantly, restoration of WNT4 expression mediated by AAV effectively promotes symmetric crypt fission and thus improves the regularity of regenerating crypts in mice with radiation-induced injury.

**Conclusions:**

Our study highlights the critical role of WNT4 in the regulation of crypt fission and provides WNT4 as a potential therapeutic target for radiation enteritis.

**Graphical Abstract:**

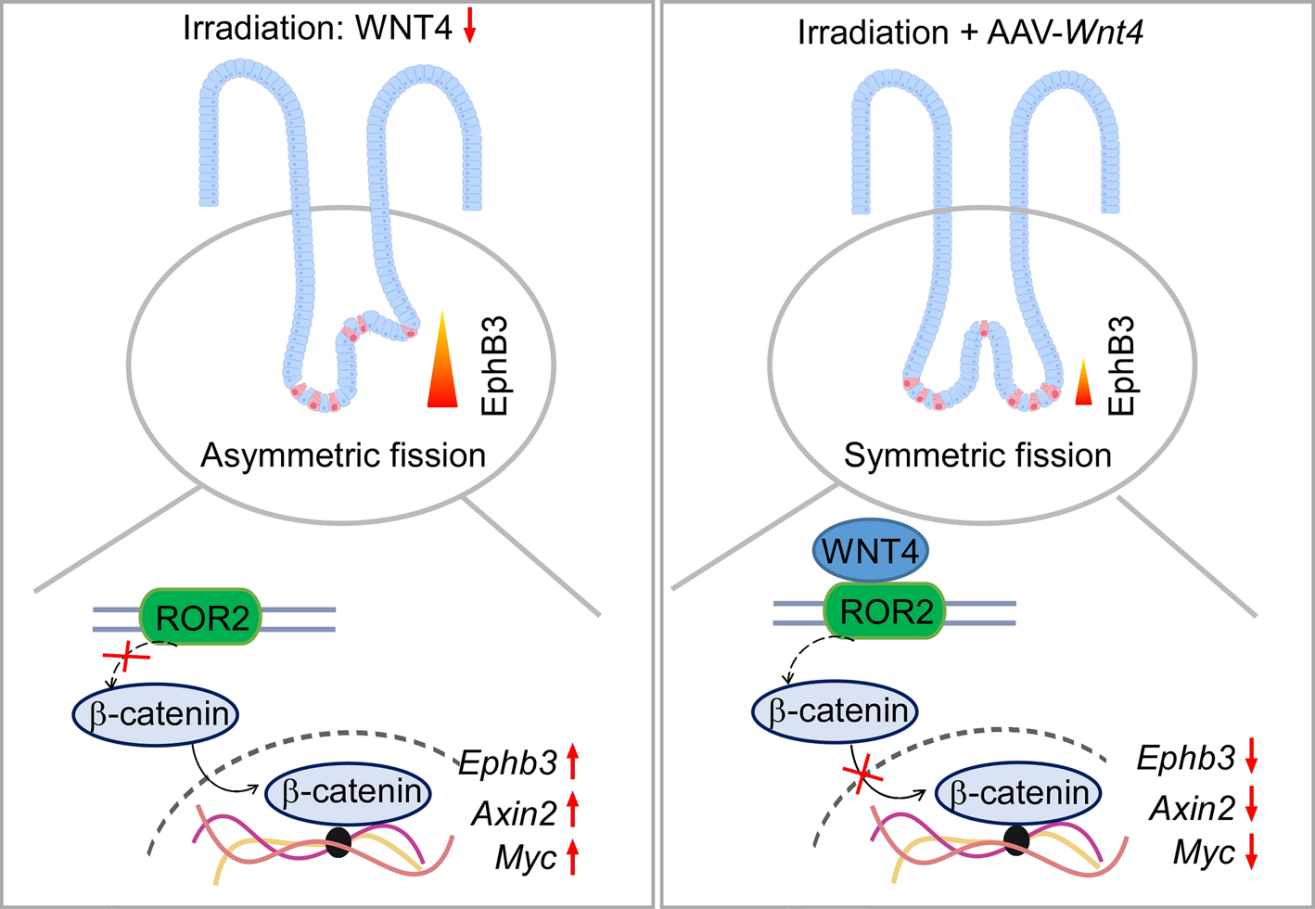

**Supplementary Information:**

The online version contains supplementary material available at 10.1186/s11658-024-00677-4.

## Introduction

The intestinal epithelium is characterized by rapid self-renewal, which occurs every 4–5 days [1, 2]. Intestinal stem cells (ISCs) located at the crypt base give rise to transit-amplifying progenitor cell population [3]. These precursors further differentiate into different cell lineages and follow a bidirectional migration path, that is, enterocytes, goblet cells, and enteroendocrine cells migrate up to the villus, while Paneth cells migrate downward and reside at the bottom of the crypt [4]. Eph/Ephrin signaling plays a central role in directing cell migration, and disruption of EphB3 signaling results in misposition of Paneth cells, which do not migrate downward but scatter along the crypt-villus axis [5, 6].

Crypt fission is an important process of intestinal tissue expansion during postnatal development [7]. The number of crypts increases in the form of fission, in which a single crypt divides into two daughter crypts [8]. The ways of crypt fission include symmetry and asymmetry [9–11]. The symmetric fission is characterized by a bifurcation appearing in the middle of the crypt base, which extends upward to form two daughter crypts of equal length, while the initial bifurcation of asymmetric fission is asymmetrically placed at the crypt base or above the crypt base, and eventually forms two branches of unequal length [12]. Intestinal organoids maintain the structural characteristics of normal intestinal epithelium, and the formation of new branches during the growth of organoids mimics the crypt fission in intestinal tissue [12, 13]. There are two cell types in the crypt base, Lgr5^+^ stem cells and Paneth cells, which play distinct roles in the fission process [12, 14]. It has been reported that the location of Paneth cells determines the initiation site of crypt fission, and the mislocation of Paneth cells drives asymmetric fission [12, 15].

The WNT family plays critical roles in intestinal epithelium [16–19]. The canonical WNT/β-catenin cascade is essential for homeostasis and regeneration of intestinal epithelium [20]. WNT/β-catenin signaling also regulates the expression of EphB3 to control the localization of Paneth cells [5]. The non-canonical WNT ligands signal via planar cell polarity (PCP) or calcium intracellular pathways, and can either activate or inhibit β-catenin pathway [21]. WNT5a has been reported to promote crypt fission in a TGF-β-dependent manner during regeneration after epithelial injury [22]. Another most widely studied typical WNT ligand, WNT4, is known to play key roles in the development and homeostasis of various organs [23]. However, whether WNT4 plays a role in intestinal epithelial regeneration, especially in the crypt fission process, is still unknown.

In clinical practice, intestinal tissue damage is inevitable when radiotherapy is performed on patients with pelvic malignant tumors [24], and the regeneration of intestinal epithelium after radiation injury largely depends on crypt fission [25]. However, how this regeneration process is regulated after radiation injury is poorly understood. Here, we applied the organoid model to reveal a particular role of WNT4 in promoting symmetric fission of crypts, and further evaluated the potential therapeutic value of adeno-associated virus (AAV)-delivered *Wnt4* overexpressing in promoting intestinal tissue repair in a radiation injury mouse model.

## Materials and methods

### Patients and samples

In total, 18 specimens were obtained from patients (female, age range from 31 to 77 years) with radiation enteritis (RE) who had received radiation therapy with a total dose of 50–98 Gy for cervical cancer, were diagnosed with RE on the basis of pelvic radiotherapy history radiological and colonoscopic features as described previously [26], and underwent surgical resection of the affected segment of intestine. Then, seven paired RE specimens were obtained from resected RE lesions and paired adjacent non-injured intestinal tissues. Specimens were snap-frozen or formalin-fixed for further use immediately after acquisition.

### Animal study

All animals were raised in specific pathogen-free conditions and maintained in a controlled temperature environment with a 12 h light/dark cycle. The radiation-induced intestinal injury mouse model was established using female C57BL/6J mice aged 8–10 weeks purchased from GemPharmatech Co., Ltd. (Nanjing, China). After anesthesia, mice received abdominal irradiation with a single dose of 10.5 Gy containing the gastrointestinal tract using a RS2000 X-ray Irradiator (Rad Source Technologies, Inc., Boca Raton, FL, USA), and the rest of the body including the thorax, head and neck, and extremities were shielded. Mice in the normal control group only received sham irradiation after anesthesia. Mice were euthanized 4 weeks after irradiation and small intestinal tissues were removed for histological examination.

For AAV treatment experiment, the mice were intraperitoneally injected with AAV-*Wnt4* (AAV-CMV-*Wnt4*-FLAG-GdGreen, OBiO Technology Co., Ltd, Shanghai, China) or AAV-vector (AAV-CMV-GdGreen, OBiO Technology Co., Ltd, Shanghai, China) at a dose of 2 × 10^11^ vg/mouse on the next day after 10.5 Gy of abdominal irradiation. At 4 weeks after irradiation, the mice were sacrificed and the small intestine tissues were collected for further examination.

We defined symmetric and asymmetric crypt fission in mouse small intestinal tissue with reference to previous report [27]. Symmetric crypt fission: bifurcated crypt showing two daughter crypts of the same length, diameter, and shape connected by a single crypt lumen. Asymmetric crypt fission: branched crypt showing two or more daughter crypts with varying diameters, lengths, and shapes connected by a single crypt lumen.

### Mouse crypt isolation and organoid culture

Mouse crypt isolation and organoid culture were performed as described previously [28]. Briefly, mouse small intestine was removed and opened longitudinally. After gently scraping the surface feces and mucus, the intestinal tissue was cut into 2-mm pieces and washed with cold phosphate buffered saline (PBS). Tissues were incubated in 5 mM ethylenediaminetetraacetic acid (EDTA) at 4 °C for 30 min. After incubation, the supernatant was removed, and the tissue pieces were resuspended in ice-cold D-PBS and vigorously triturated using a 25-ml pipette. The separated crypts in the supernatant were filtered through a 70-μm filter into a new tube [precoated with 1% bovine serum albumin (BSA)] and then centrifuged at 500 *g* for 5 min. The pellet crypts were then resuspended in Matrigel (BD Biosciences, San Jose, CA, USA) and transferred to 24-well cell culture plate. Mouse IntestiCult™ Organoid Growth Medium (Stemcell Technologies) was added after the Matrigel was polymerized and changed every 2 days.

### Lentivirus transfection

For the preparation of lentiviral particles,*Wnt4*-overexpressing plasmid (pEZ-Lv242-*Wnt4*, GeneCopoeia, Maryland, USA) or the control plasmid (pEZ-Lv242-vector, GeneCopoeia, Maryland, USA) were transfected in HEK 293FT cells together with lentiviral packaging vectors using PEI transfection solution. Supernatants containing virus were collected 48 and 72 h after transfection and centrifuged at 50,000 *g* for 90 min. The viral pellets were then resuspended in 500 μl of organoid culture medium supplemented with 10 μg/ml polybrene.

For lentiviral transduction of organoids, organoids were harvested by mechanically disrupting the Matrigel, and then digested with trypsin for 3 min at 37 °C. After removal of trypsin by centrifugation, the organoids were mixed with the virus solution and centrifuged on 32 °C at 600 g for 1 h in a 48-well plate to enhance transduction efficacy. The organoid-virus mixture was incubated in a culture incubator for 1 h at 37 °C, and then resuspended with 500 μl of organoid culture medium and centrifuged for 5 min at 500 g to pellet organoids. The organoid pellet was then embedded in 50 μl of ice-cold matrigel and cultured in 0.5 ml of mouse IntestiCult™ Organoid Growth Medium.

### siRNA transfection

siRNA transfection of organoids was performed according to previous study [29]. The siRNA transfection solution was prepared by mixing *Wnt4* siRNA (RiboBio Co. Ltd, Guangzhou, China) or control siRNA (RiboBio Co. Ltd, Guangzhou, China) with RNAiMAX (Invitrogen) in DMEM (Gibco) containing 10% FBS (Gibco), and was added directly to organoids after 2 days of culture in a 24-well plate. The medium was replaced with fresh organoid culture medium 48 h after transfection.

### Histology, immunohistochemistry (IHC), and immunofluorescence (IF)

Human and mouse intestinal tissues were fixed with 4% paraformaldehyde and paraffin embed. Sections were stained with hematoxylin and eosin (HE).

IHC staining was carried out using SP-9000 SPlink Detection Kit (ZSGB-Bio, Beijing, PR China) following the manufacturer’s protocol. The following primary antibodies were used: anti-WNT4 (1:100, 14371-1-AP, Proteintech group), anti-Ki67 (1:300, ab16667, Abcam) and anti-lysozyme (1:300, ab108508-40, Abcam) antibodies.

For IF staining of tissue sections, permeabilization was performed with PBS containing 0.1% Triton X-100. After blocking with 1% BSA, the sections were incubated with primary antibodies at 4 °C overnight, washed and subsequently incubated with fluorescence-conjugated secondary antibodies Alexa Fluor 488 (1:1000, A-32723, Invitrogen) or Alexa Fluor 594 (1:1000, A-11037, Invitrogen) at room temperature for 1 h. Nuclei were counterstained with DAPI (Beyotime, Beijing, China). Confocal laser scanning was performed using a Leica TCS-SP8 confocal microscope (Mannheim, Baden-Wuerttemberg, Germany). The primary antibodies utilized in this study were anti-EphB3 (1:300, AF432-SP, R&D systems), anti-lysozyme (1:300, ab108508-40, Abcam), anti-WNT4 (1:100, 14371-1-AP, Proteintech group), anti-Flag (1:200, F1804, Sigma), and anti-ROR2 (1:8, Ror2, DSHB) antibodies.

For IF staining of organoids, organoids isolated from Matrigel were fixed with 4% paraformaldehyde at 37 °C for 45 min, permeabilized using 1% Triton-X100 for 1 h, and then incubated with blocking buffer (3% normal goat serum, 1% BSA and 0.2% Triton-X100 in PBS) for 1 h at room temperature. The following primary antibodies were used to incubate overnight with the organoids in Working Buffer (0.3% normal goat serum, 0.1% BSA, 0.2% Triton-X100 in PBS): anti-lysozyme (1:200, ab108508-40, Abcam), anti-Ki67 (1:200, ab16667, Abcam), anti-WNT4 (1:50, 14371-1-AP, Proteintech group), and anti-ROR2 (1:4, Ror2, DSHB) antibodies. After washing, AlexaFluor-conjugated secondary antibodies (1:1000, Invitrogen) along with Phalloidin (1:100, PF00003, Proteintech group) were applied overnight in Working Buffer. Nuclei were counterstained with DAPI (1:5000, Beyotime, Beijing, China) or Hoechst 33342 (1:5000, Beyotime, Beijing, China). After mounting with ProLong Gold antifade (Meilunbio), images were taken with Lecia TCS-SP8 or STELLARIS 8 FALCON confocal microscopes (Mannheim, Baden-Wuerttemberg, Germany).

### Time series of live imaging of organoid culture

Live imaging of organoids was captured using Operetta CLS HCS (PerkinElmer, USA). Imaging processing was conducted using the Harmony 4.8 software (PerkinElmer, USA).

### 3D image processing and analysis

Organoids were imaged with a Lecia STELLARIS 8 FALCON confocal microscope (Mannheim, Baden-Wuerttemberg, Germany) using 40× objective lens, and Z stacks were taken at 1 μm steps. Measurements of crypt length and Paneth cell distance were based on 3D projections of organoids using ImageJ software (National Institutes of Health, Bethesda, MD, USA). Symmetric fission in organoids was assessed according to previous report [12]. That is, a ratio of the length of the shorter to the length of the longer daughter crypt greater than 0.75 was considered a symmetric fission. The localization of Paneth cells was determined on the basis of staining for lysozyme, and the distance of each Paneth cell from the crypt base was measured to analyze their distribution.

### RT-qPCR

Total RNA in tissues or organoids was isolated using Trizol reagent (Invitrogen, Carlsbad, CA). Reverse transcription was carried out using ReverTra Ace Master Mix (TOYOBO, Osaka, Japan). Real-time quantitative polymerase chain reaction (RT-qPCR) was conducted with FastStart Essential DNA Green Master (Roche, Basel, Switzerland) using RT-PCR systems (Applied LightCycler96 Roche). *GAPDH* or *ACTB* served as the internal control. The primer sequences applied in this study are listed in Additional file 1, Supplementary Table 1.

### Immunoprecipitation (IP)

IP assay was performed according to our previous study [28]. Briefly, whole small intestinal tissues from normal C57BL/6J mice (8–10 weeks, female) were lysed using lysis buffer (150 mM NaCl, 10 mM HEPES, pH 7.4, 1% NP-40). Lysates were precleaned with 25 μl of protein G affinity gel (E3403, Sigma, St Louis, MO, USA) at 4 °C for 1 h. The supernatant was immunoprecipitated with 1 μg of anti-ROR2 antibody (Ror2, DSHB) or mouse immunoglobulin G (IgG) isotype control (MAB002, R&D systems) at 4 °C overnight, and then incubated with 30 μl of protein G gel at 4 °C for 1 h. The immunoprecipitated complex was washed with wash buffer (150 mM NaCl, 10 mM HEPES, pH 7.4, 0.1% NP-40) six times, and eluted with 2× sample buffer, and then subjected to western blot analysis.

### In situ hybridization (ISH)

The probe for *Wnt4* was designed and synthesized by Servicebio (Wuhan, China). The probe sequences were as follows: 5′-TGAGTTTCTCGCACGTCTCCTCTT-3′, 5′-TCCACAAAGGACTGTGAGAAGGCTAC-3′, 5′-GGCTTTAGATGTCTTGTTGCACGTGC-3′.

Small intestines from mice at 4 weeks post-irradiation (10.5 Gy) and the normal control mice were fixed in formalin, embedded in paraffin and sliced into 4 μm sections. After being dewaxed and rehydrated, the sections were repaired in a citric acid antigen retrieval solution (pH 6.0) at 90 °C for 48 min and digested in proteinase K (20 μg/ml) at 40 °C for 10 min. After blocking endogenous peroxidase with 3% methanol –H_2_O_2_, sections were prehybridized at 37 °C for 1 h and hybridized in a solution containing the probes overnight at 40 °C. Sections were washed in 2 × SSC for 10 min at 37 °C, in 1 × SSC at 37 °C for 2 × 5 min and in 0.5 × SSC at room temperature for 10 min. Sections were then blocked in normal rabbit serum at room temperature for 30 min and incubated with anti-DIG-HRP at 37 °C for 50 min. After washing for several times with PBS, color development was performed with DAB and nuclei were counterstained with hematoxylin. Signal intensities in the crypt and villus regions were quantified separately with four random 200× magnification fields per slide using ImageJ software.

### Statistical analysis

Data analyses were performed using GraphPad Prism 8 (GraphPad, San Diego, CA, USA). Unpaired or paired two-sided Student’s *t*-tests were used to calculate differences between two groups. one-way or two-way analysis of variance (ANOVA) with Tukey’s multiple comparisons test were applied to compare multiple groups. Pearson correlation coefficient test was used to analyze the relationship between variables. Bars represent mean ± SD. *P* < 0.05 was considered significant. All experiments were carried out at least three times.

## Results

### WNT4 suppresses crypt regeneration in organoids

**Fig. 1:**
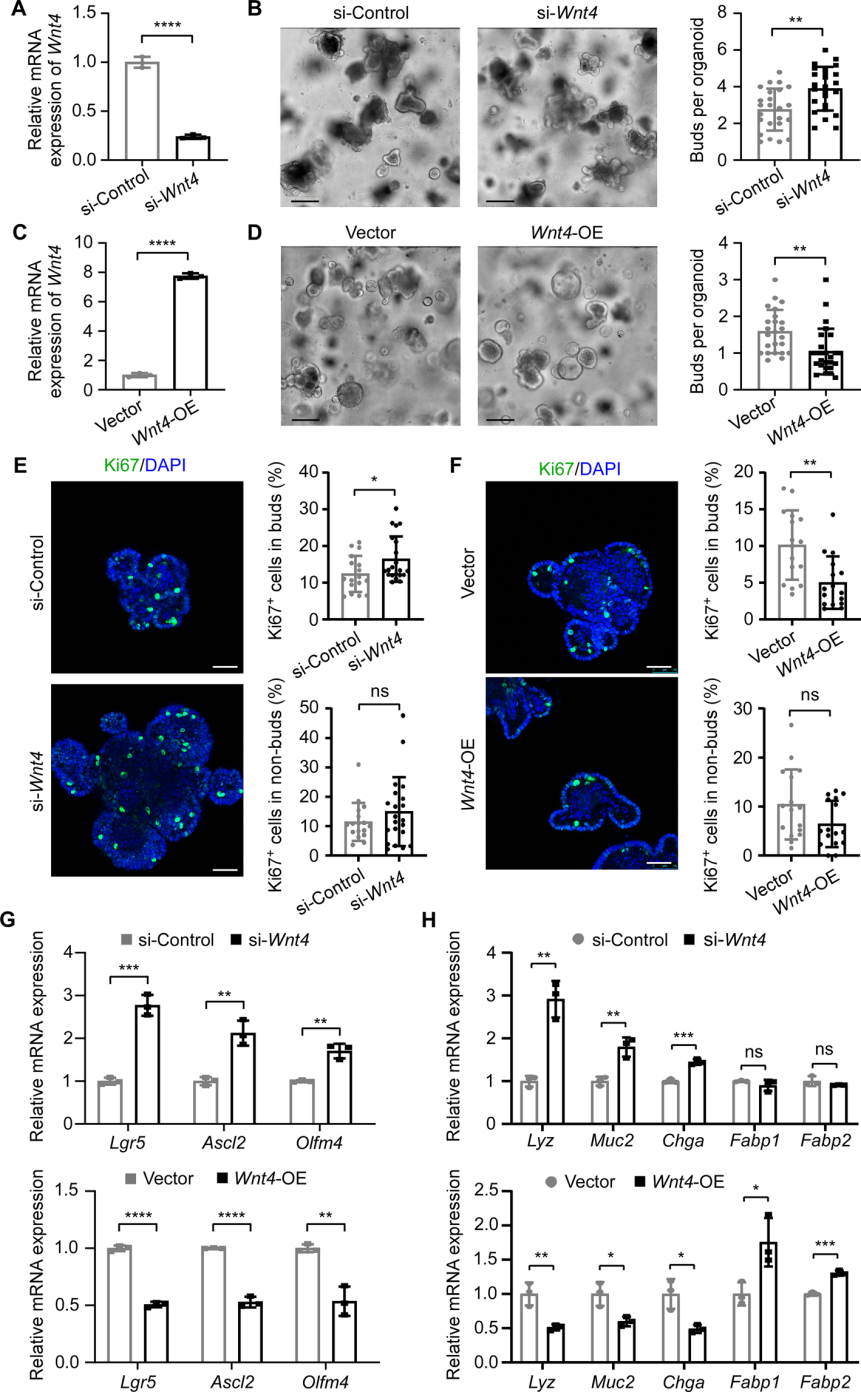
WNT4 suppresses intestinal organoid branching. **A** RT-qPCR analysis of the interference efficiency of *Wnt4* by siRNA in mouse small intestinal organoids. **B** Representative images (left) and budding numbers (right) of mouse small intestinal organoids transfected with either control siRNA (si-control) or *Wnt4* siRNA (si-*Wnt4*) after 60 h. Scale bars, 200 μm. **C** RT-qPCR analysis of the overexpression efficiency of *Wnt4* in mouse small intestinal organoids. **D** Representative images (left) and budding numbers (right) of mouse small intestinal organoids transfected with either vector control (vector) or *Wnt4*-overexpressing construct (*Wnt4*-OE) after 60 h. Scale bars, 200 μm. **E** Representative images of IF staining for Ki67 in mouse small intestinal organoids transfected with either control siRNA or *Wnt4* siRNA. Scale bars, 50 μm. The percentages of Ki67-positive cells in the bud and non-bud areas of each organoid (*n* = 17 for si-control and *n* = 21 for si-*Wnt4*) were quantified separately. **F** Representative images of IF staining for Ki67 in mouse small intestinal organoids transfected with either vector control or *Wnt4*-overexpressing construct. Scale bars, 50 μm. The percentages of Ki67-positive cells in the bud and non-bud areas of each organoid (*n* = 16 for both Vector and *Wnt4*-OE) were quantified separately. **G** RT-qPCR analysis of the mRNA levels of stem cell marker genes (*Lgr5*, *Ascl2* and *Olfm4*) in mouse small intestinal organoids after knockdown or overexpression of *Wnt4*. **H** RT-qPCR analysis of the mRNA levels of cell differentiation markers (*Lyz*, *Muc2*, *Chga*, *Fabp1* and *Fabp*2) in mouse small intestinal organoids after knockdown or overexpression of *Wnt4*. All values are means ± SD. Statistical analyses were performed by unpaired Student’s *t*-test (**A**–**H**). ns, not significant, *p < 0.05, **p < 0.01, ***p < 0.001, ****p < 0.0001

Previous study reported that the formation of new branches in organoid mimics the process of crypt fission in tissue [12, 13], therefore, we used organoid culture model to examine the effect of WNT4 on crypt fission. The results showed that knockdown of *Wnt4* promoted the branching of organoids, whereas overexpression of *Wnt4* suppressed the branch formation of organoids (Fig. 1A–D). Moreover, the number of Ki67-positive cells increased in *Wnt4*-knockdown organoids while decreased in *Wnt4*-overexpressing organoids, in both the bud and non-bud areas, compared with their respective controls, although the non-bud areas did not reach statistical significance (Fig. 1E, F), suggesting that WNT4 inhibits the proliferation of organoids. Furthermore, RT-qPCR analysis showed that the expression levels of ISC markers *Lgr5*, *Ascl2* and *Olfm4* were increased in *Wnt4*-knockdown organoids while decreased in *Wnt4*-overexpressing organoids, compared with their respective controls (Fig. 1G). We also examined the effect of WNT4 on cell differentiation. The results showed that knockdown of *Wnt4* significantly upregulated, while overexpression of *Wnt4* downregulated the expression of *Lyz*, *Muc2* and *Chga*. Although *Wnt4* knockdown did not significantly alter the levels of *Fabp1* and *Fabp2*, *Wnt4* overexpression markedly increased the expression of *Fabp1* and *Fabp2*, suggesting that WNT4 might inhibit the differentiation of secretory epithelial cells but promote the differentiation of absorptive cells (Fig. 1H). Collectively, these results indicate that WNT4 suppresses crypt regeneration via inhibiting crypt fission, ISC activity, cell proliferation, and differentiation.

### WNT4 promotes symmetric fission of crypt in organoids

**Fig. 2:**
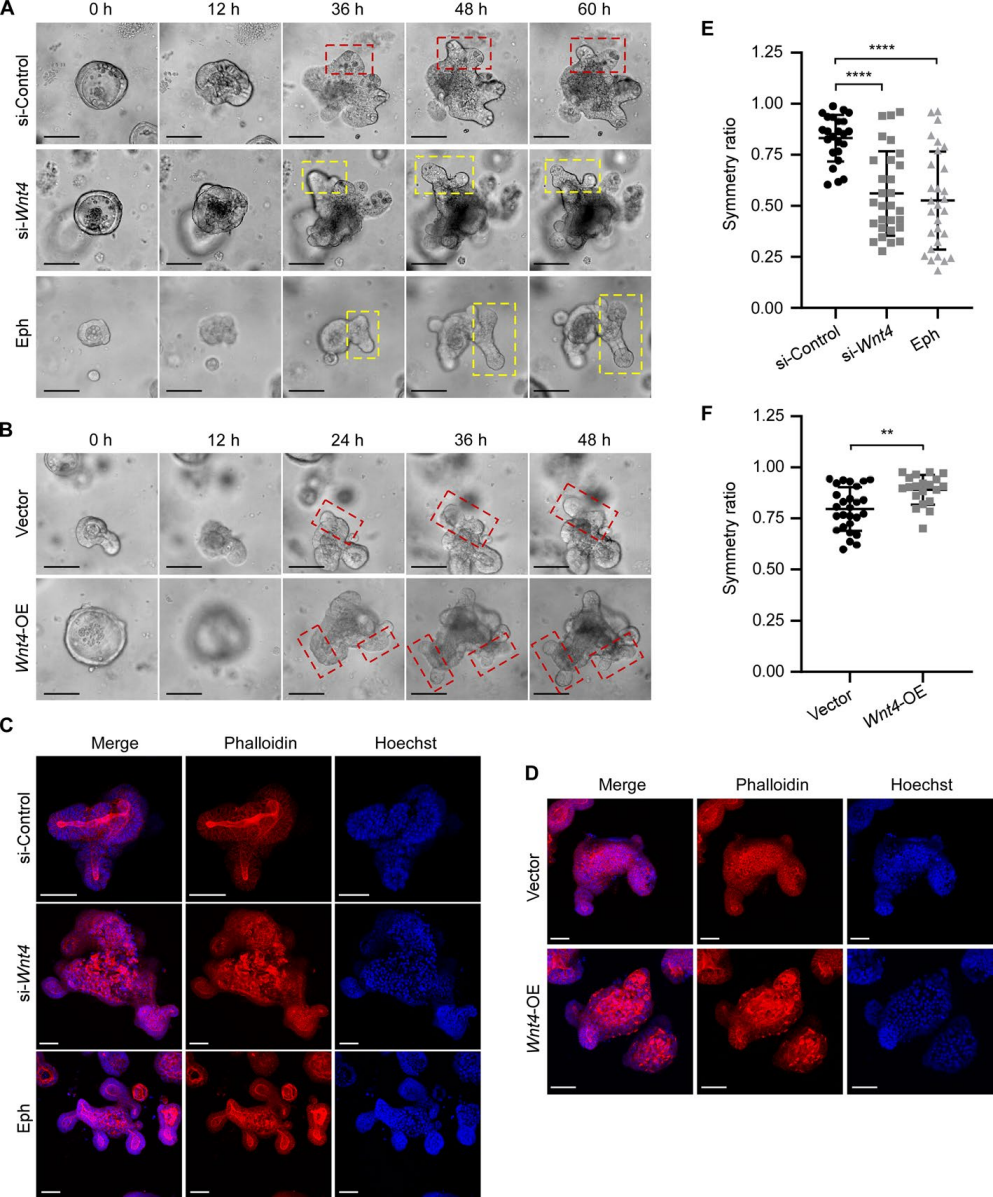
WNT4 promotes symmetric fission of crypt in organoids. **A**, **B** Representative time-lapse images of mouse small intestinal organoids transfected with si-control, si-*Wnt4*, Vector or *Wnt4*-OE, or treated with an inhibitory Eph fragment (Eph, 10 μg/ml) at the indicated time points. Scale bars, 100 μm. Red dashed boxes indicate symmetric fissions, and yellow dashed boxes indicate asymmetric fissions. **C**, **D** Representative 3D projections of organoids stained against F-actin (Phalloidin, red) and nuclei (Hoechst, blue) after transfection with si-control, si-*Wnt4*, Vector or *Wnt4*-OE, or treated with an inhibitory Eph fragment (Eph, 10 μg/ml). Scale bars, 50 μm. **E**, **F** The symmetry ratio was calculated by dividing the length of the short daughter crypt by the length of the long daughter crypt based on the 3D projections of organoids in **C** and **D**. *n* = 24 for si-control, *n* = 29 for si-*Wnt4*, *n* = 29 for Eph, *n* = 27 for vector, and *n* = 20 for *Wnt4*-OE. All values are means ± SD. Statistical analyses were performed by one-way ANOVA with Tukey’s multiple comparisons test (**E**) or unpaired Student’s *t*-test (**F**). **p < 0.01, ****p < 0.0001

We further investigated whether WNT4 regulates the symmetry of crypt fission. After transfection of *Wnt4* siRNA or overexpression vector, we dynamically monitored the organoid fission process using time-lapse imaging (Fig. 2A, B). Since Eph/Ephrin signaling controls the localization of Paneth cells, which in turn determines the initiation site of crypt fission, the inclusion of inhibitory Eph fragments could lead to more asymmetric fissions. Therefore, we took Eph-treated organoids as positive controls for asymmetric fission. To more accurately analyze the effect of WNT4 on the symmetry of the crypt fission, we compared the length of the two daughter crypts on the basis of 3D structure of the organoids stained against F-actin and nuclei being visualized using confocal microscopy (Fig. 2C, D). We found that the frequency of asymmetric fission increased after suppressing *Wnt4* expression (Fig. 2E). Conversely, overexpression of *Wnt4* increased the symmetric fissions of the organoids compared with the vector control (Fig. 2F). These results indicate that WNT4 promotes symmetric crypt fission in organoids.

### WNT4 promotes the correct localization of Paneth cells at the crypt base

**Fig. 3:**
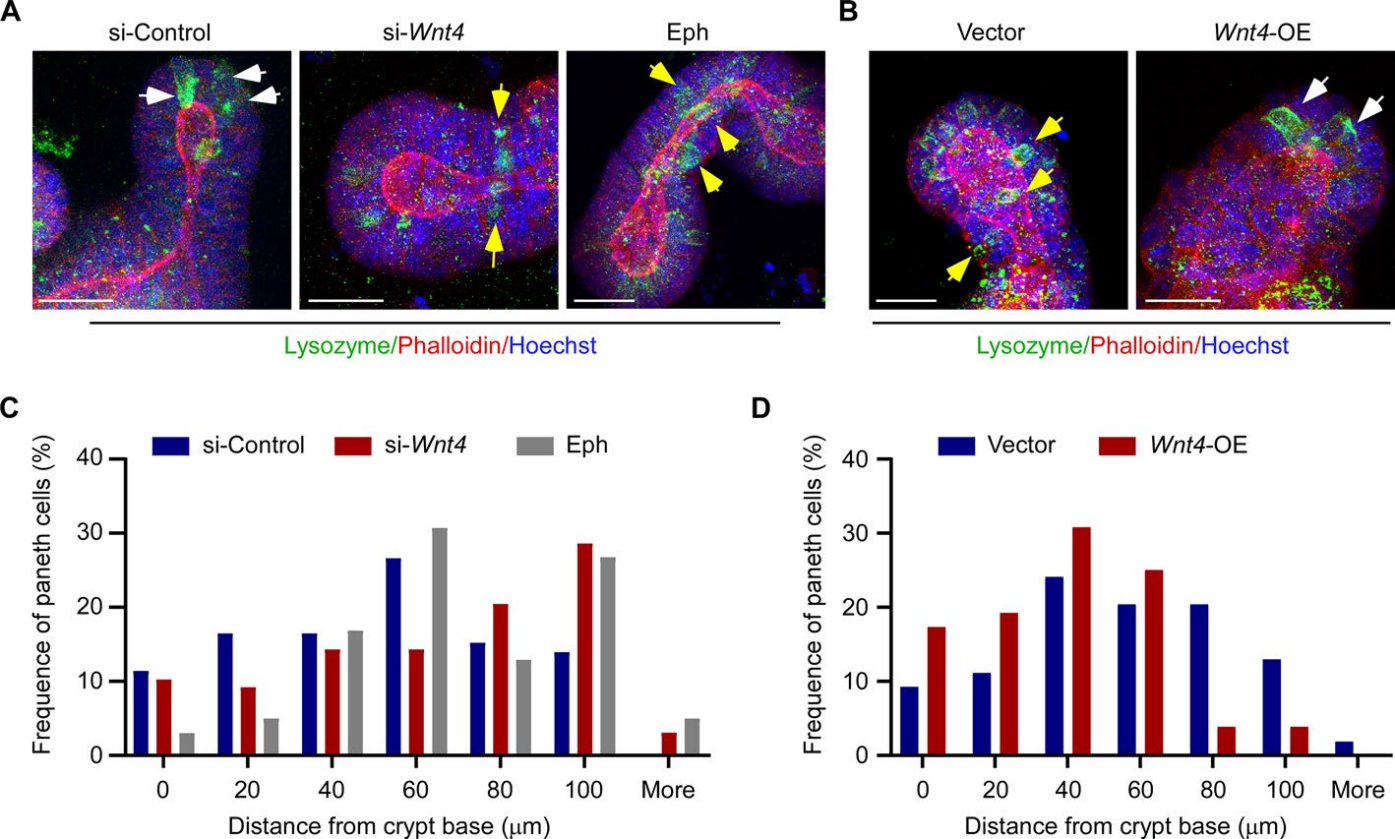
WNT4 promotes the correct localization of Paneth cells in the crypt. **A**,** B** 3D projections of mouse small intestinal organoids stained to visualize Paneth cells (lysozyme, green), F-actin (Phalloidin, red), and nuclei (Hoechst, blue). Organoids were transfected with si-control or si-*Wnt4*, or treated with an inhibitory Eph fragment (Eph, 10 μg/ml) in **A**, or transfected with Vector or *Wnt4*-OE in **B**. Scale bars, 25 μm. White arrows indicate Paneth cells positioned in the crypt base and yellow arrows indicate Paneth cells positioned away from the crypt base. **C**, **D** The frequency of Paneth cells distanced from the crypt base was quantified on the basis of the 3D projections of organoids in **A** and **B**. The tip of the organoid bud was identified as the crypt base. *n* = 79 Paneth cells for si-control, *n* = 98 Paneth cells for si-*Wnt4*, *n* = 101 Paneth cells for Eph, *n* = 54 Paneth cells for Vector, and *n* = 52 Paneth cells for *Wnt4*-OE

The localization of Paneth cells can affect the symmetry of crypt fission [12]. We next examined whether WNT4 expression affects Paneth cell localization. The 3D projections of organoids stained against lysozyme to visualize Paneth cells revealed that, compared with the control siRNA, knockdown of *Wnt4* expression resulted in more Paneth cells being mispositioned away from the crypt base (Fig. 3A, C). Meanwhile, overexpression of *Wnt4* led more Paneth cells to localization near the crypt base (Fig. 3B, D). In addition, WNT4 also reduced the total number of Paneth cells in organoid (Additional file 1: Supplementary Fig. 1), in line with the result mentioned above that WNT4 inhibited LYZ expression (Fig. 1H). Collectively, these results suggest that WNT4 may influence the symmetric fission of crypts by regulating the localization of Paneth cells.

### WNT4 negatively regulates canonic WNT/β-catenin pathway and depends on ROR2 to promote symmetric fission

**Fig. 4:**
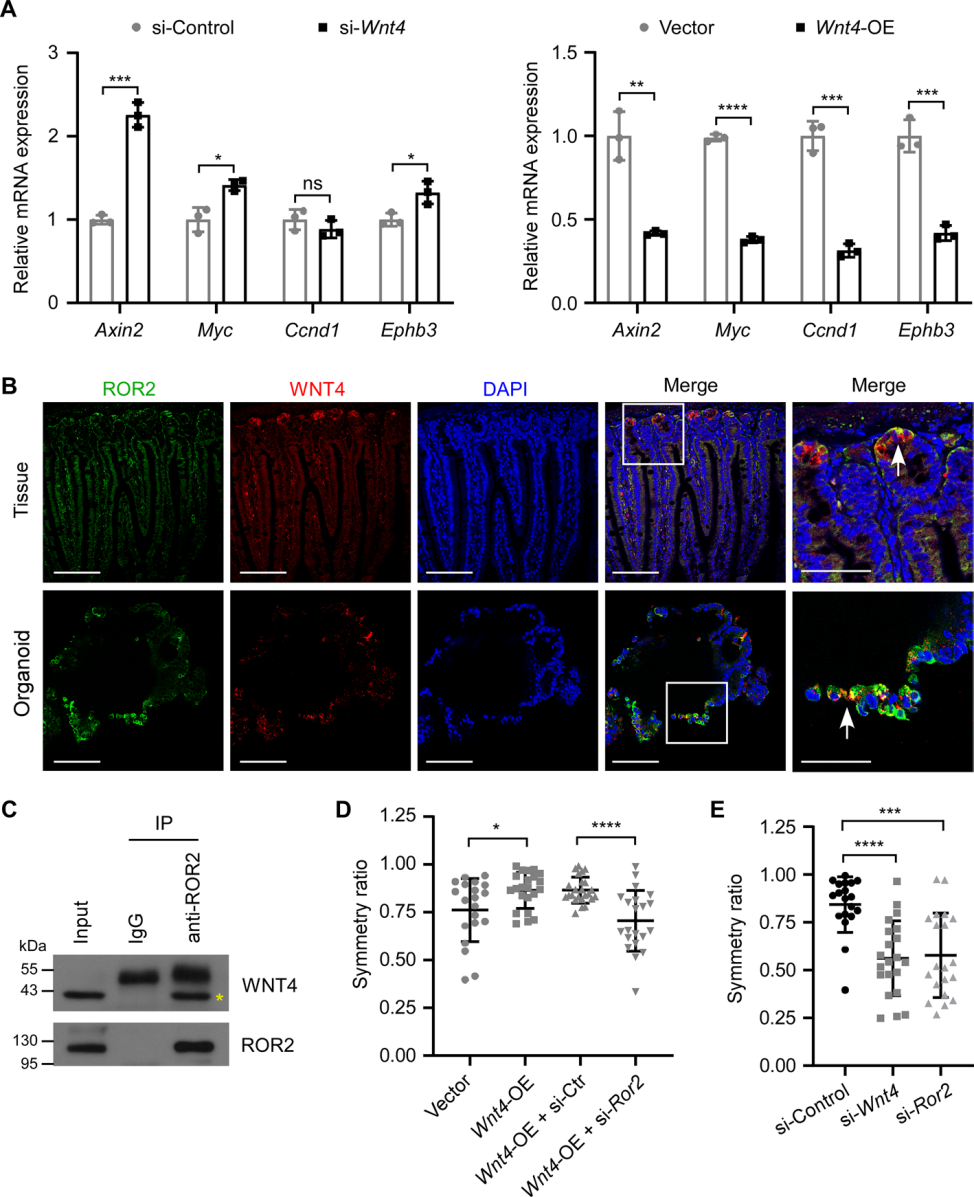
WNT4 negatively regulates WNT/β-catenin signaling, and relies on ROR2 receptor to promote symmetric crypt fission. **A** RT-qPCR analysis of the expression changes of WNT/β-catenin target genes (*Axin2*, *Myc*, *Ccnd1* and *Ephb3*) in mouse small intestinal organoids after knockdown (left) or overexpression (right) of *Wnt4*. **B** Colocalized expression of ROR2 (green) and WNT4 (red) in both normal mouse small intestinal tissue and organoid was detected by IF staining. Boxed regions are magnified and shown in the right column. Nuclei were counterstained with DAPI (blue). White arrows indicate colocalization. Scale bars, 100 μm (columns 1–4), 50 μm (column 5). **C** Lysate from the whole mouse small intestine was immunoprecipitated with anti-ROR2 or anti-IgG and analyzed by western blot with anti-WNT4 and anti-ROR2 antibodies. Yellow asterisk indicates the WNT4 band. **D** The symmetry ratio was calculated by dividing the length of the short daughter crypt by the length of the long daughter crypt on the basis of the 3D projections of mouse small intestinal organoids with overexpression of *Wnt4* combined with or without knockdown of *Ror2* (*n* = 20 for vector, *n* = 22 for *Wnt4*-OE, *n* = 24 for *Wnt4*-OE + si-Ctr, *n* = 21 for *Wnt4*-OE + si-*Ror2*). **E** The symmetry ratio was calculated by dividing the length of the short daughter crypt by the length of the long daughter crypt on the basis of the 3D projections of mouse small intestinal organoids with either knockdown of *Wnt4* or knockdown of *Ror2* (*n* = 19 for si-control, *n* = 21 for si-*Wnt4*, *n* = 20 for si-*Ror2*). All values are means ± SD. Statistical analyses were performed by unpaired Student’s *t*-test between two groups (**A**), and one-way ANOVA with Tukey’s multiple comparisons test (**D**, **E**). ns, not significant, *p < 0.05, **p < 0.01, ***p < 0.001, ****p < 0.0001

Previous studies have reported that β-catenin/TCF signaling regulates the localization of epithelial cells by controlling the expression of EphB/EphrinB [5]. Therefore, we further investigated whether WNT4 mediates the canonic WNT/β-catenin signaling pathway. The results showed that knockdown of *Wnt4* significantly increased, while overexpression of *Wnt4* decreased the mRNA levels of β-catenin target genes *Axin2* and *c-Myc* in the organoids (Fig. 4A). Moreover, knockdown of *Wnt4* also up-regulated, while overexpression of *Wnt4* suppressed the mRNA level of *Ephb3* in the organoids (Fig. 4A). These data suggest that WNT4 negatively regulates the canonic WNT/β-catenin signaling pathway and EphB3 expression.

Since WNT5a inhibits canonic WNT/β-catenin signaling through receptor tyrosine kinase-like orphan receptor ROR2 [30, 31], we hypothesized that WNT4 might also function through ROR2. IF staining showed that WNT4 and ROR2 were both positively expressed in the crypt and co-localized in the epithelial cells in both normal mouse small intestinal tissue and organoid (Fig. 4B). In addition, co-IP assay showed that WNT4 was able to bind to ROR2 in mouse normal small intestine (Fig. 4C). We further investigated whether WNT4 regulates the symmetry of crypt fission depending on ROR2. The results showed that inhibition of *Ror2* expression greatly abrogated the promoting effect of *Wnt4* overexpression on the symmetric fission of crypt in organoids (Fig. 4D, Additional file 1: Supplementary Fig. 2). Moreover, knockdown of *Ror2* had a similar effect as knockdown of *Wnt4* in promoting asymmetric crypt fission of organoids (Fig. 4E). These data suggest that WNT4 promotes symmetric fission depending on ROR2.

### WNT4 expression is decreased in the radiation-injured intestinal tissue

**Fig. 5:**
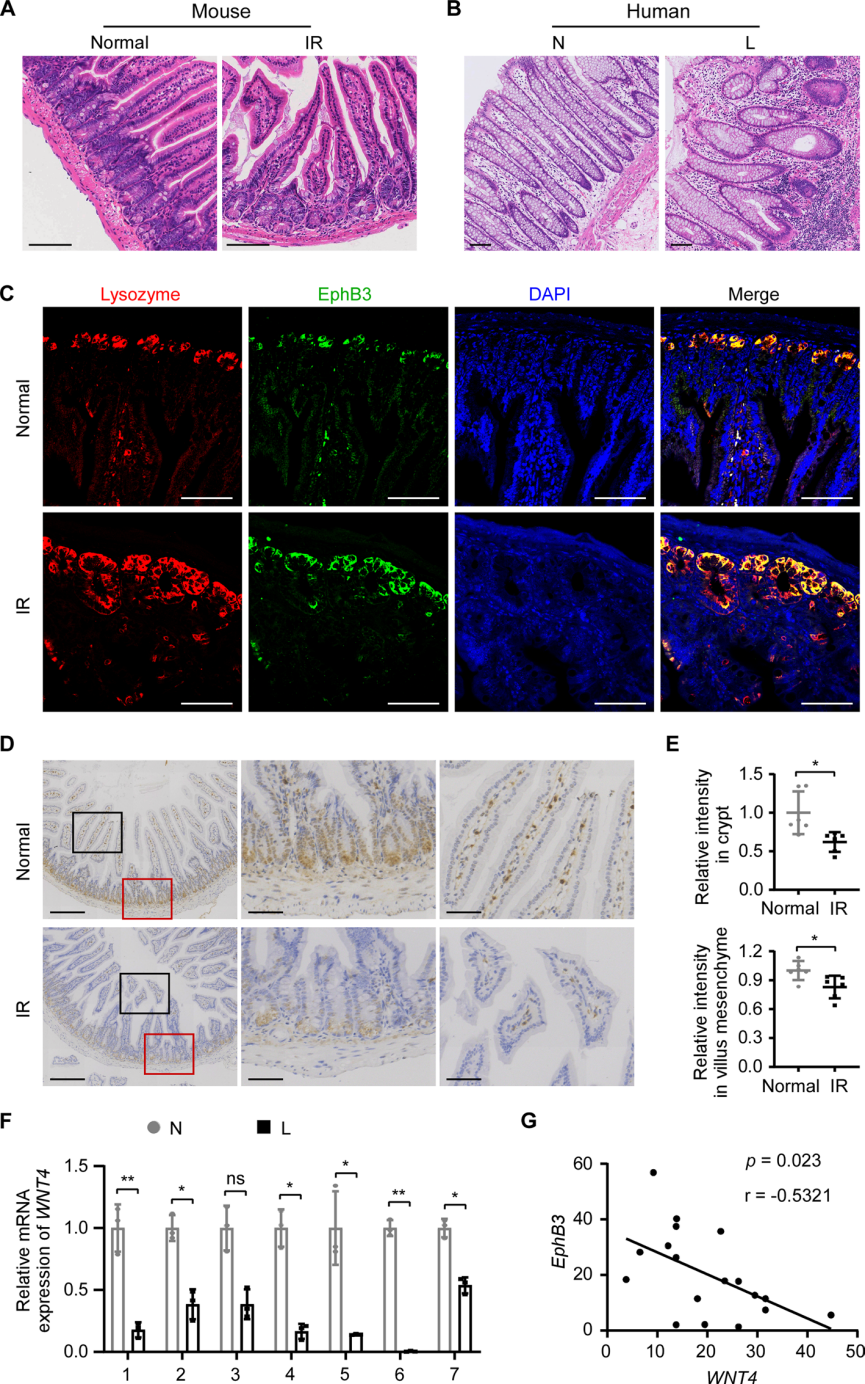
WNT4 expression is decreased in the radiation-injured human and mouse intestinal tissues. **A** HE staining of the small intestines of normal control mouse (Normal) and mouse with 10.5 Gy of abdominal irradiation after 4 weeks (IR). Scale bars, 100 μm. **B** HE staining of the radiation-injured lesion (L) and paired adjacent non-injured intestinal tissue (N) in RE patient. Scale bars, 100 μm. **C** IF staining was performed using anti-lysozyme (labeled Paneth cells, red) and anti-EphB3 (green) antibodies in the small intestinal tissues of normal and IR mice. Nuclei were counterstained with DAPI (blue). Scale bars, 100 μm. **D** ISH analysis of *Wnt4* expression in the small intestinal tissues of normal and IR mice. The crypt regions (red boxes) were magnified in the middle column, and the villous regions (black boxes) were magnified in the right column. Scale bars, 200 μm (left column) and 50 μm (middle and right columns). **E** The intensity of *Wnt4* expression in the crypts and villus mesenchyme in the normal (*n* = 6) and IR (*n* = 6) mice was quantified using ImageJ software based on the ISH analysis in **D**. **F** RT-qPCR analysis of *WNT4* expression in seven clinical samples of RE lesions (L) and paired adjacent non-injured intestinal tissues (N). **G** Correlation analysis between the relative *WNT4* mRNA level and *EphB3* mRNA level in the intestinal lesions of patients with RE (*n* = 18). Data are means ± SD. Statistical analysis was performed by unpaired Student’s *t*-test (**E**), paired Student’s *t*-test (**F**), and Pearson correlation coefficient test (**G**). ns, not significant, *p < 0.05, **p < 0.01

Through histological examination of RE specimens of patients and animal models, we found that compared with normal intestinal crypts, which were closely arranged in “rows of test tubes” perpendicular to the muscularis mucosae, the regenerated crypts in the radiation-injured intestine usually showed irregular morphology and size, which we speculate may be partly the result of asymmetric fission of crypts during regeneration (Fig. 5A, B). Meanwhile, lysozyme-positive Paneth cells were confined to the base of crypts and colocalized with EphB3 in the mouse normal small intestine; while in the irradiation group, lysozyme-positive and EphB3-positive areas exceeded the base of crypts and expanded upward, which means that Paneth cells were mislocalized and EphB3 expression was increased (Fig. 5C). Furthermore, ISH analysis showed that *Wnt4* expression was positive in the crypt and villus mesenchyme of the normal mice, but was decreased in both the crypt and villus mesenchyme of the irradiated mice (Fig. 5D, E). In addition, IHC staining and RT-qPCR analysis further confirmed that WNT4 expression was decreased in the irradiated mouse small intestine, compared with the normal control (Additional file 1: Supplementary Fig. 3A, B). We further examined WNT4 expression in human RE specimens. RT-qPCR analysis showed that *WNT4* expression was lower in the lesions of RE than in their paired adjacent non-injured intestinal tissues (Fig. 5F). Moreover, *WNT4* expression was negatively correlated with *EphB3* expression in human RE (Fig. 5G). Taken together, these results support the hypothesis that decreased expression of WNT4 causes excessive expression of EphB3, which leads to the misposition of Paneth cells and thus the asymmetric fission of crypts during radiation-induced crypt regeneration.

### WNT4 supplementation mediated by AAV promotes the symmetric fission of crypts after irradiation in mice

**Fig. 6:**
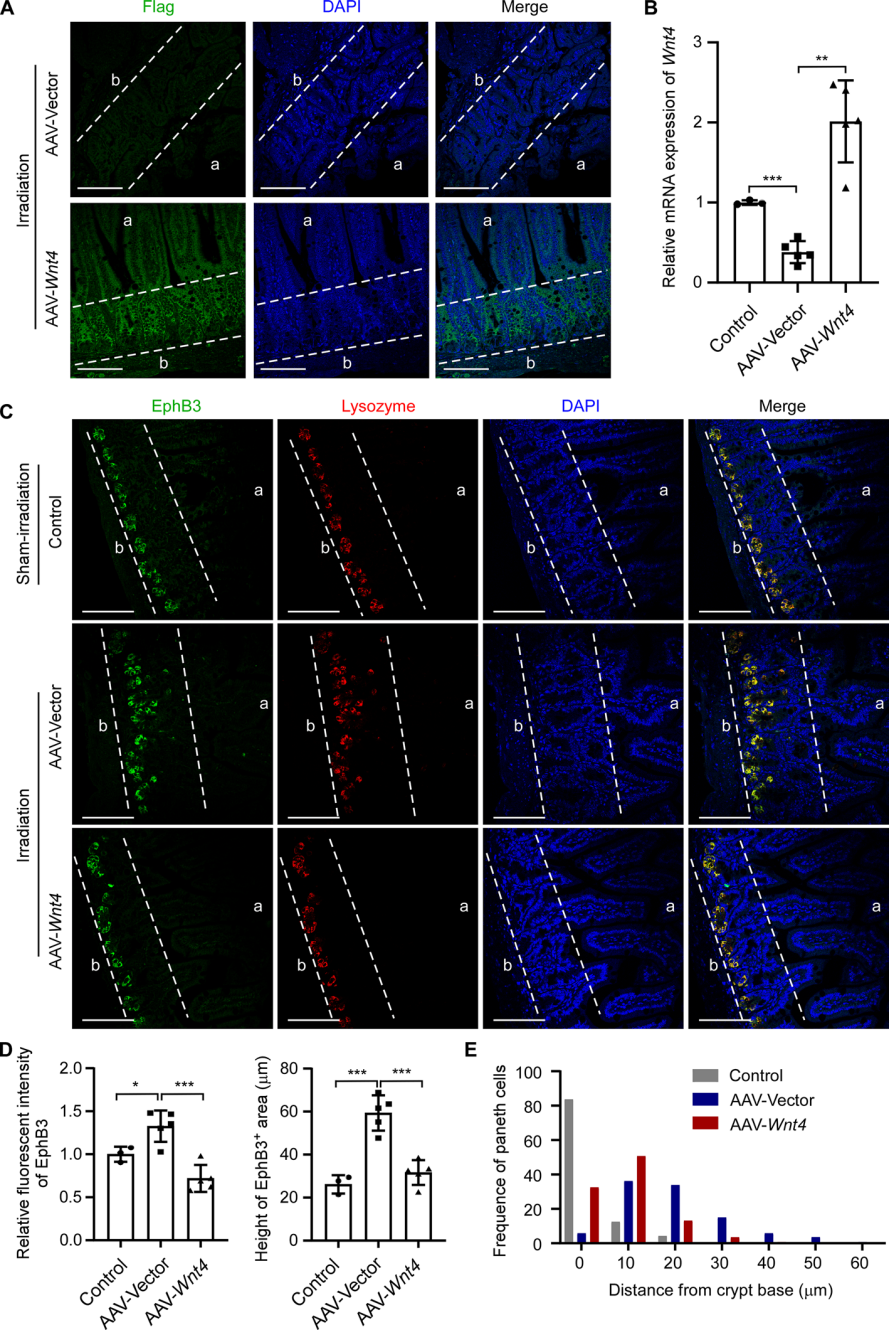
AAV-mediated WNT4 supplementation inhibits EphB3 expression in small intestinal crypts in mice with radiation injury. The mice received 10.5 Gy of abdominal irradiation or sham-irradiation as control. The next day, AAV-vector or AAV-*Wnt4* was intraperitoneally injected into the irradiated mice. At 4 weeks after irradiation, the small intestinal tissues were collected and analyzed. **A** The overexpression of WNT4 in the small intestines of irradiated mice treated with AAV-*Wnt4* was confirmed by IF staining with anti-Flag antibody (green). Nuclei were counterstained with DAPI (blue). Scale bars, 100 μm. Dashed lines indicate crypt area. a: apical; b: basal. **B** RT-qPCR analysis of *Wnt4* expression in the entire small intestinal tissues of the irradiated mice treated with AAV-vector (*n* = 5) or AAV-*Wnt4* (*n* = 5) or the sham-irradiated control mice (*n* = 3). **C** IF staining was performed using anti-lysozyme (labeled Paneth cells, red) and anti-EphB3 (green) antibodies in the small intestinal tissues of the three groups of mice. Nuclei were counterstained with DAPI (blue). Scale bars, 100 μm. Dashed lines indicate crypt area. a: apical; b: basal. **D** The intensity of EphB3 expression and the height of the EphB3-positive area were quantified using ImageJ on the basis of the IF staining of EphB3 in **C**. **E** The frequency of Paneth cells distanced from the crypt base was quantified using ImageJ on the basis of the IF staining of lysozyme in **C**. Values are means ± SD. Statistical analyses were performed by one-way ANOVA with Tukey’s multiple comparisons test (**B**, **D**). *p < 0.05, **p < 0.01, ***p < 0.001

We next explored whether *Wnt4* overexpression delivered by AAV could promote more symmetric fission of the crypts in a mouse radiation-injury model. AAV-*Wnt4* or AAV-vector was injected intraperitoneally the following day after 10.5 Gy of abdominal irradiation. At 4 weeks after irradiation, crypt regeneration in small intestine was assessed. IF staining of anti-Flag showed that WNT4 was successfully overexpressed in the small intestinal epithelium in the irradiated AAV-*Wnt4*-treated group (Fig. 6A). RT-qPCR analysis also showed that, compared with the sham-irradiated control group, *Wnt4* mRNA expression was significantly decreased in the small intestine of the irradiated AAV-vector-treated mice, while it was greatly restored in the irradiated AAV-*Wnt4* group (Fig. 6B). IF staining showed that, compared with the sham-irradiated control group, the intensity of EphB3 expression and the height of the EphB3-positive area were increased in the irradiated AAV-vector group, whereas these were largely decreased in the irradiated AAV-*Wnt4* group (Fig. 6C, D). Furthermore, in the irradiated AAV-vector group, more Paneth cells were located far from the crypt base, whereas in the irradiated AAV-*Wnt4* group, more Paneth cells were distributed near the crypt base (Fig. 6C, E).

**Fig. 7:**
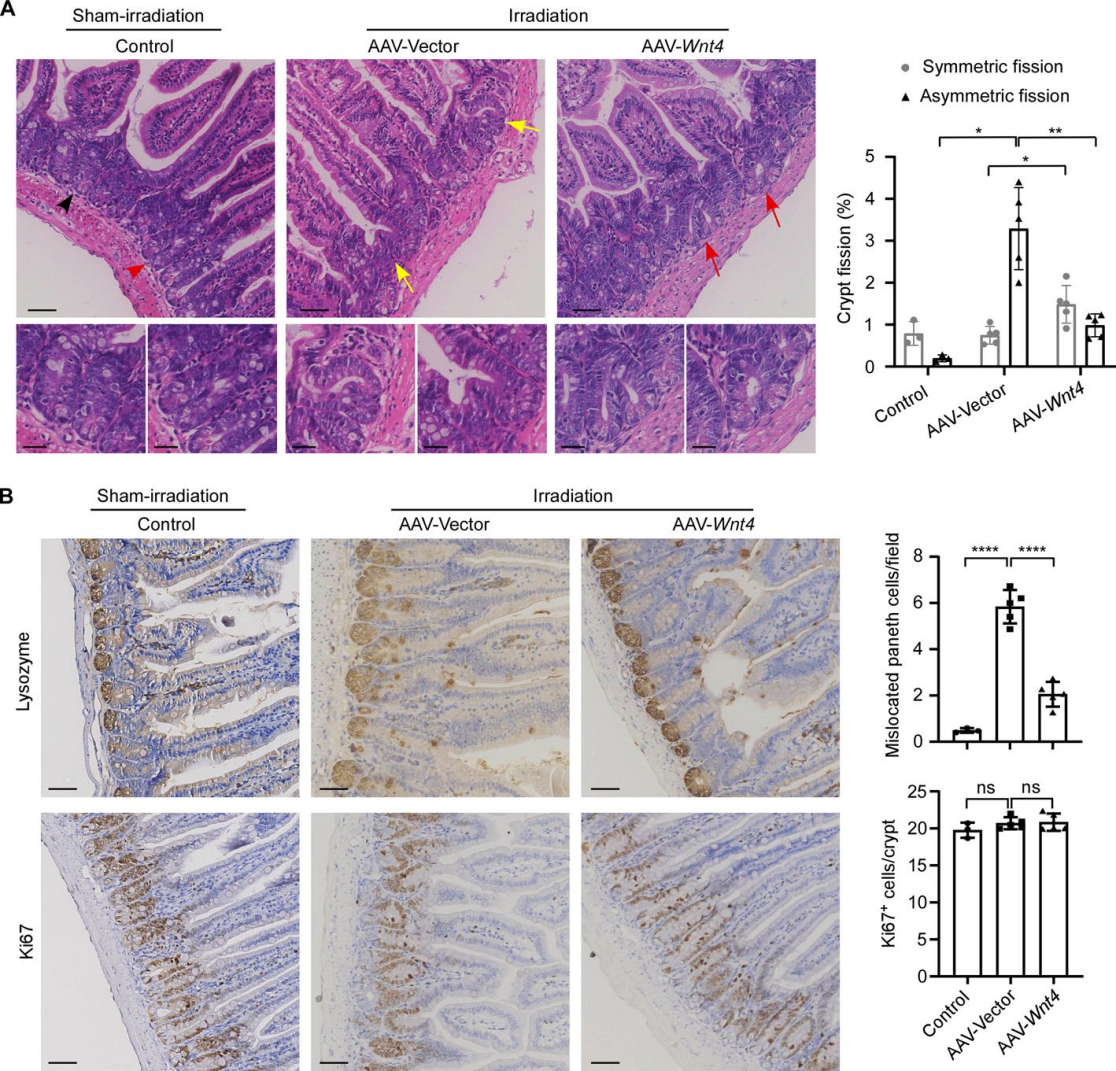
AAV-mediated WNT4 supplementation reduces asymmetric fission of small intestinal crypts in mice with radiation injury. **A** Representative images of HE staining (left) of the small intestinal tissues of the irradiated mice treated with AAV-vector (*n* = 5) or AAV-*Wnt4* (*n* = 5) or the sham-irradiated control mice (*n* = 3). The proportions of symmetric and asymmetric crypt fissions in the small intestinal tissues of mice in the three groups were calculated (right). The black and red arrow heads in the sham-irradiated control group indicate crypt not undergoing fission and crypt undergoing symmetric fission, respectively, which are magnified in the next row (left). The yellow arrows in the irradiated AAV-vector group indicate crypts undergoing asymmetric fissions, which are magnified in the next row (middle). The red arrows in the irradiated AAV-*Wnt4* group indicate crypts undergoing symmetric fissions, which are magnified in the next row (right). Scale bars, 50 μm (upper row) and 25 μm (lower row). **B** Representative images of lysozyme and Ki67 staining of the small intestinal tissues of the irradiated mice treated with AAV-vector (*n* = 5) or AAV-*Wnt4* (*n* = 5) or the sham-irradiated control mice (*n* = 3). Scale bars, 50 μm. The numbers of mislocated Paneth cells per 20 × field and Ki67^+^ cells per crypt were quantified. All values are means ± SD. Statistical analyses were performed by two-way ANOVA (**A**) or one-way ANOVA (**B**) with Tukey’s multiple comparisons test. ns, not significant. *p < 0.05, **p < 0.01, ****p < 0.0001

We further calculated the proportions of crypts with symmetric or asymmetric fission in the small intestines in each treatment groups on the basis of HE staining of tissue sections (Fig. 7A). Our results showed that symmetric fission occasionally occurred and asymmetric fission was much rarer in normal mouse small intestine (Fig. 7A), which is similar to the findings in colon [32, 33]. However, in the irradiated AAV-vector-treated mice, asymmetric fission was significantly increased (Fig. 7A), which further supports our previous observation that the size and morphology of the regenerated crypts were irregular after irradiation (Fig. 5A, B). Importantly, restoration of WNT4 expression after irradiation in AAV-*Wnt4*-treated mice resulted in a significant decrease in asymmetric fission, while a concomitant increase in symmetric fission was observed (Fig. 7A). Moreover, IHC staining showed that the number of mislocated Paneth cells was significantly increased in the small intestines of irradiated mice with AAV-vector treatment compared with the sham-irradiated control mice, while it was decreased in the irradiated AAV-*Wnt4*-treated mice (Fig. 7B). In addition, there was no significant difference in the number of Ki67-positive cells per crypt among these groups (Fig. 7B). Taken together, these results suggest that restoration of WNT4 expression could promote the correct localization of Paneth cells, reduce asymmetric fission of crypts, and ultimately improve the regularity of the reconstructed intestinal epithelium after radiation injury.

## Discussion

In intestinal tissues after radiation injury, it is often observed that the size and shape of the regenerated crypts are irregular, disordered, and the normal arrangement pattern of the intestinal epithelium is disrupted, which may be related to excessive asymmetric fission of crypts during regeneration. Asymmetric crypt fission that occurs in inflammatory bowel disease is considered to be a kind of pathological aberration with a tendency to develop mucosal dysplasia [32]. However, little is known about the mechanisms regulating the symmetry of crypt fission. In this study, we found that WNT4 expression is inhibited in the intestinal tissue after radiation injury. Using organoid culture model, we further discovered that WNT4 promotes the symmetric fission of crypts by regulating the canonic WNT/β-catenin signaling pathway. Importantly, WNT4 supplementation effectively promotes more symmetric crypt fissions in a radiation-injury animal model, improving the regularity of the reconstructed intestinal epithelium.

Although it was reported that the incidence of symmetric and asymmetric fission is similar during normal intestinal development [12], it seems that the regeneration of crypts after radiation injury is prone to more asymmetric fissions, which may be partially owing to low crypt density, high fission frequency, and uneven mechanical pressure from the surrounding environment. Indeed, in colorectal adenomas and ulcerative colitis, a high frequency of crypt fission events is often accompanied by a higher rate of asymmetric fission, whereas in normal adult colonic mucosa, crypt fission rarely occurs [9, 11, 32, 34, 35]. However, on the basis of our findings, we propose that another important factor contributing to the increased asymmetric fission is the altered expression of certain key molecules that regulate crypt fission, such as decreased expression of WNT4. Using the organoid culture system and an animal model of RE, our study provides evidence that decreased WNT4 is responsible for increased EphB3, mislocalized Paneth cells, and excessive asymmetric crypt fission in the radiation-injured small intestine. A high proportion of asymmetric and irregular crypts will cause the intestinal mucosa to lose its normal morphological structure, which may cause the disorder of intestinal function.

The Eph receptor tyrosine kinases mediate cell repulsion and compartmentalization during embryonic development [36, 37]. When engaged with their ephrin ligands, the bidirectional signal is conveyed in both the Eph-expressing (forward signaling) and the ephrin-expressing (reverse signaling) cells [38]. In the intestinal epithelium, EphB/ephrinB signaling dictates cell positioning and ordered migration [5]. In the adult normal intestine, EphB and ephrinB expression are controlled by β-catenin signaling, and their expression patterns show inverse gradients along the crypt-villus axis, that is, high expression of EphB3 is restricted to the crypt base, while ephrinB1 expression is strongly detected at the crypt-villus junction and gradually decreases toward the crypt bottom [5]. EphB3 deletion or blocking EphB3 signaling results in Paneth cells not being located at the crypt base, but randomly spreading throughout the crypt [5, 6]. Alternatively, the upregulation of EphB3 expression owing to constitutive β-catenin signaling activation also leads to mislocalization of Paneth cells [39–41], suggesting that disrupting the expression gradient of EphB3 could impair the directed migration of Paneth cells. Our study demonstrated that the decreased expression of WNT4 in radiation-injuried intestinal epithelium leads to overactivation of β-catenin pathway and increase of EphB3, with EphB3 not being confined to the crypt base, but expanding upward, thus causing the misposition of Paneth cells. This was further supported by our in vivo evidence that WNT4 supplementation promotes restoration of the normal gradient of EphB3 and proper localization of Paneth cells in the animal model.

WNT ligands can selectively activate either the canonic β-catenin signaling or the non-canonic pathways largely depending on the availability of their receptors. For example, WNT5a can activate the non-canonic JNK signaling and inhibit canonic WNT/β-catenin signaling through ROR2 [30, 31, 42, 43]; on the contrary, WNT5a amplified canonic β-catenin signaling when the Frizzled (Fzd) receptor Fzd4 is expressed [31]. Studies have demonstrated that WNT4 could regulate the canonic β-catenin signaling and non-canonic PCP/JNK and Ca^2+^/CaMK pathways via interacting with Fzds [23, 44, 45]. However, whether WNT4 relies on ROR receptors to activate downstream signaling in regulating certain cellular processes is rarely reported. Our study demonstrated that ROR2 is expressed in the crypt epithelial cells and interacts with WNT4. Moreover, disrupting the expression of ROR2 eliminates the promotion effect of WNT4 on symmetric fission of crypt, suggesting that ROR2 might be a bona-fide functional receptor of WNT4.

## Conclusions

Our study demonstrates that WNT4 suppresses the expression of EphB3, which is elevated in RE, through inhibition of β-catenin pathway, thereby promoting the correct localization of Paneth cells as well as the symmetric fission of small intestinal crypts. Overexpression of WNT4 effectively promotes the symmetric fission of small intestinal crypts and thus improves the regularity of the regenerating crypts in mice with radiation-induced injury, suggesting that WNT4 could be an attractive therapeutic target for RE.

## Supplementary Information

Additional file 1: Supplementary Fig. 1. WNT4 inhibits the differentiation of Paneth cells. Supplementary Fig. 2. WNT4 promotion of symmetric crypt fission is dependent on the ROR2 receptor. Supplementary Fig. 3. WNT4 expression is decreased in radiation-injured intestinal tissues of mice. Supplementary Table 1. Primer sequences for RT-qPCR.

Additional file 2. Raw images of IP.

## Data Availability

The data supporting this study are available from the corresponding author upon reasonable request.

## Abbreviations

ISCs: Intestinal stem cells
AAV: Adeno-associated virus
RE: Radiation enteritis
PCP: Planar cell polarity
IHC: Immunohistochemistry
IF: Immunofluorescence
HE: Hematoxylin and eosin
IP: Immunoprecipitation
ISH: In situ hybridization
Fzd: Frizzled
