# Supplementary material for: WNT4 promotes the symmetric fission of crypt in radiation-induced intestinal epithelial regeneration
Source: Cell Mol Biol Lett. 2024 Dec 26;29:158. doi: 10.1186/s11658-024-00677-4 (PMC11670417; doi:10.1186/s11658-024-00677-4)
Supplement: Supplementary file 1 — Additional file 1: Supplementary Fig. 1. WNT4 inhibits the differentiation of Paneth cells. Supplementary Fig. 2. WNT4 promotion of symmetric crypt fission is dependent on the ROR2 receptor. Supplementary Fig. 3. WNT4 expression is decreased in radiation-injured intestinal tissues of mice. Supplementary Table 1. Primer sequences for RT-qPCR. [file 11658_2024_677_MOESM1_ESM.docx]

**Supplementary figures**

**
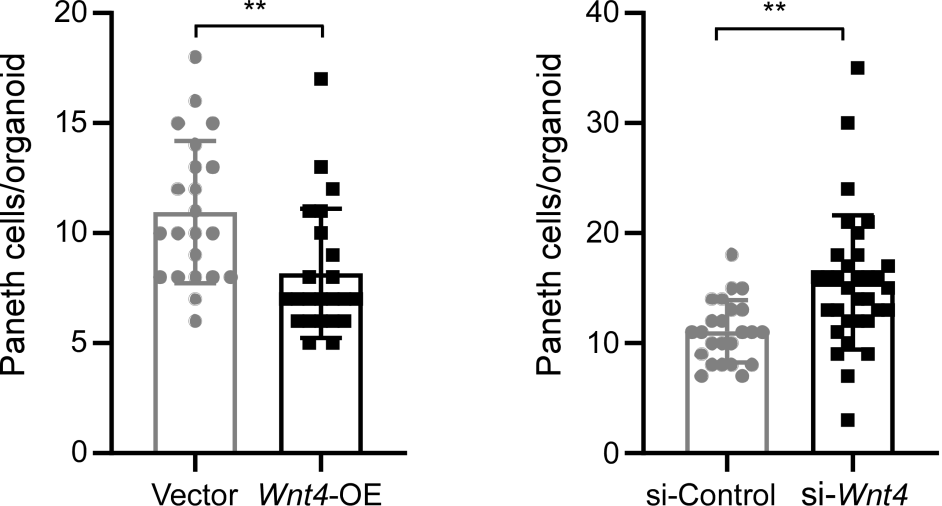
**

**Supplementary Fig. 1** WNT4 inhibits the differentiation of Paneth cells. The total number of Paneth cells per organoid was counted on the basis of 3D projections of organoids transfected with Vector (n = 22), *Wnt4*-OE (n = 23), si-Control (n = 24) or si-*Wnt4* (n = 33). All values are means ± SD. Statistical analyses were performed by unpaired Student’s *t*-test. ***p* < 0.01.

**
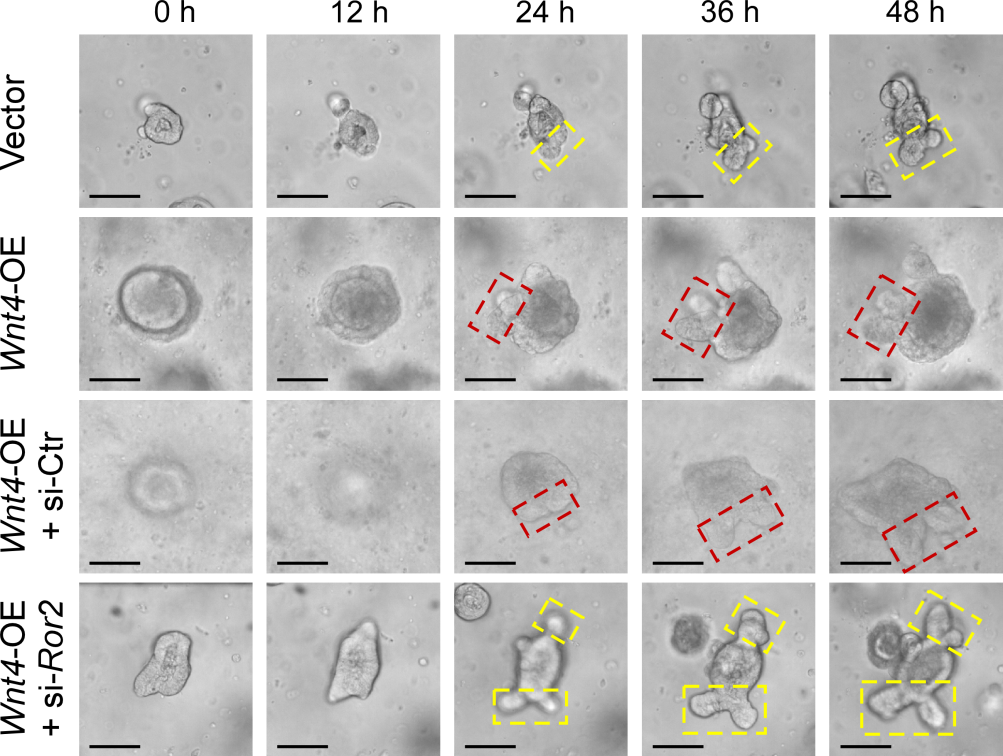
**

**Supplementary Fig. 2** WNT4 promotion of symmetric crypt fission is dependent on the ROR2 receptor. Representative time-lapse images of mouse small intestinal organoids with overexpression of *Wnt4* combined with or without knockdown of *Ror2*. Scale bars, 100 μm. Red dashed boxes indicate symmetric fissions, and yellow dashed boxes indicate asymmetric fissions.


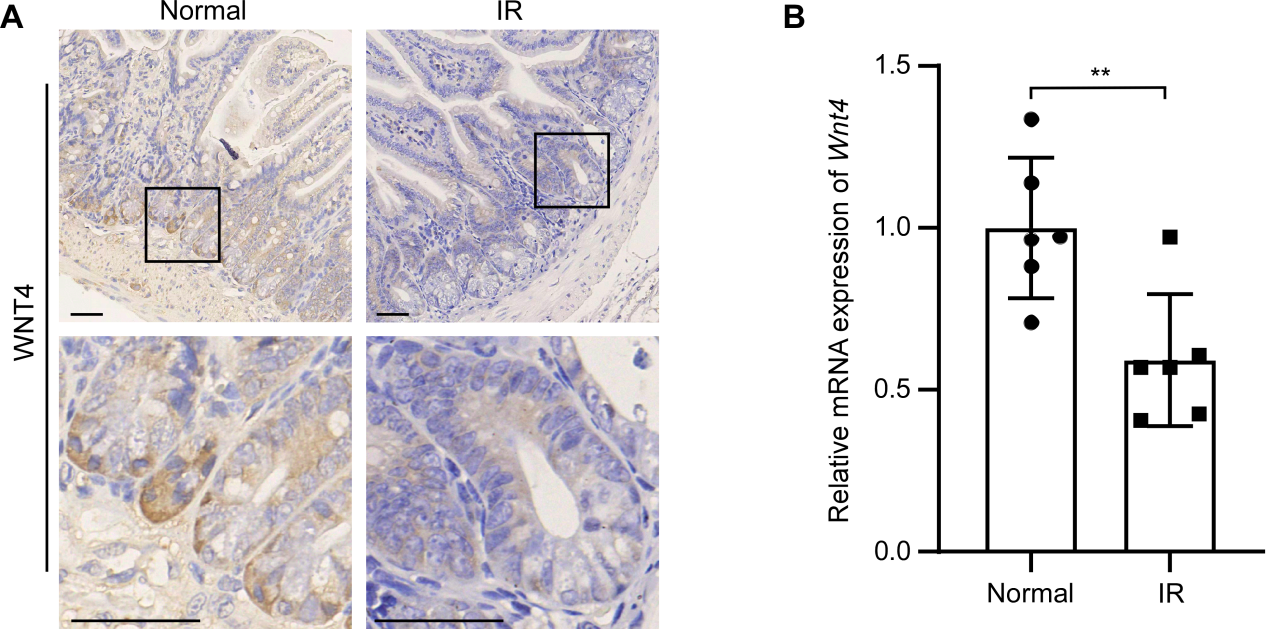


**Supplementary Fig. 3** WNT4 expression is decreased in radiation-injured intestinal tissues of mice. **A** IHC staining of WNT4 in the small intestinal tissues of normal control mouse (Normal) and mouse with 10.5 Gy of abdominal irradiation after 4 weeks (IR). Boxed regions are shown at higher magnification in the next row. Scale bars, 50 μm. **B** RT-qPCR analysis of *Wnt4* expression in the entire small intestinal tissues of Normal and IR mice (n = 6 for each group). Data are means ± SD. Statistical analysis was performed by unpaired Student’s *t*-test. ***p* < 0.01.

**Supplementary Table 1.** Primer sequences for RT-qPCR.

| Gene target | Sequence (5’ – 3’) | |
| --- | --- | --- |
| *Wnt4*-Mus | Forward | CGTGCGAGAAACTCAAAGGC |
|  | Reverse | TCCGGAACTGGTATTGGCAC |
| *Lgr5*- Mus | Forward | GGACCAGATGCGATACCGC |
|  | Reverse | CAGAGGCGATGTAGGAGACTG |
| *Ascl2*- Mus | Forward | AAGCACACCTTGACTGGTACG |
|  | Reverse | AAGTGGACGTTTGCACCTTCA |
| *Olfm4*- Mus | Forward | CAGCCACTTTCCAATTTCACTG |
|  | Reverse | GCTGGACATACTCCTTCACCTTA |
| *Ephb3*- Mus | Forward | CAAGACGCTGAAGGTGGGAT |
|  | Reverse | TGTCCGTCATTGAGCCGTAG |
| *Ccnd1*-Mus | Forward | CAACTTCCTCTCCTGCTACCG |
|  | Reverse | TGGAGGGGGTCCTTGTTTAG |
| *Axin2*-Mus | Forward | ATGAGTAGCGCCGTGTTAGTG |
|  | Reverse | GGGCATAGGTTTGGTGGACT |
| *Myc*-Mus | Forward | CCCTATTTCATCTGCGACGAG |
|  | Reverse | GAGAAGGACGTAGCGACCG |
| *Lyz1*-Mus | Forward | GGTCTACAATCGTTGTGAGTTG |
|  | Reverse | TGAGCTAAACACACCCAGTCA |
| *Muc2*-Mus | Forward | GTCCTGACCAAGAGCGAACA |
|  | Reverse | ACAGCACGACAGTCTTCAGG |
| *Chga*-Mus | Forward | AGGGGACACCAAGGTGATGA |
|  | Reverse | AGCAGATTCTGGTGTCGCAG |
| *Fabp1*-Mus | Forward | TCCGCAATGAGTTCACCCTG |
|  | Reverse | GCTTGACGACTGCCTTGACT |
| *Fabp2*-Mus | Forward | TGGAAAGGAGCTGATTGCTGT |
|  | Reverse | TCCTTCATATGTGTAGGTCTGGAT |
| *Gapdh*-Mus | Forward | AAGAAGGTGGTGAAGCAG |
|  | Reverse | TCATACCAGGAAATGAGC |
| *Actb*-Mus | Forward | GCAGATGTGGATCAGCAAGC |
|  | Reverse | GCAGCTCAGTAACAGTCCGC |
| *WNT4*-Homo | Forward | TCGTCTTCGCCGTCTTCTCA |
|  | Reverse | TGACTTCCAGGTTCCGCTTG |
| *EphB3*-Homo | Forward | CAGATGACGGCAGAAGACCT |
|  | Reverse | AGGCAGCGTCTGGTTCATC |
| *ACTB*-Homo | Forward | TTGTTACAGGAAGTCCCTTGCC |
|  | Reverse | ATGCTATCACCTCCCCTGTGTG |
