# Supplementary figures and images for: WNT4 promotes the symmetric fission of crypt in radiation-induced intestinal epithelial regeneration
Source: Cell Mol Biol Lett. 2024 Dec 26;29:158. doi: 10.1186/s11658-024-00677-4 (PMC11670417; doi:10.1186/s11658-024-00677-4)

Raw image for Fig. 4C

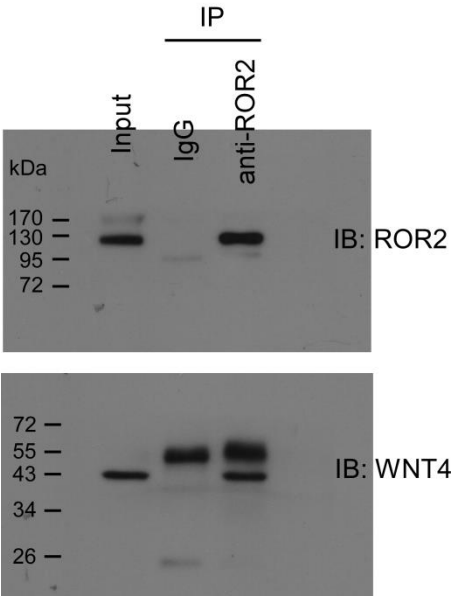

Supplement: Supplementary file 2 — Additional file 2. Raw images of IP. [file 11658_2024_677_MOESM2_ESM.pdf]
